# Patient’s Scar Satisfaction after Conventional Thyroidectomy for Differentiated Thyroid Cancer

**DOI:** 10.3390/jpm13071066

**Published:** 2023-06-29

**Authors:** Massimo Campagnoli, Valeria Dell’Era, Maria Silvia Rosa, Fabiola Negri, Eric Malgrati, Massimiliano Garzaro, Paolo Aluffi Valletti

**Affiliations:** ENT Department Maggiore della Carità Hospital, 28100 Novara, Italy; valeria.dellera@gmail.com (V.D.); m.silviarosa@gmail.com (M.S.R.); 20007648@studenti.uniupo.it (F.N.); 20008363@studenti.uniupo.it (E.M.); massimiliano.garzaro@uniupo.it (M.G.);

**Keywords:** differentiated thyroid carcinoma (DTC), scar, satisfaction, thyroidectomy, quality of life, QoL, ENT, head and neck, cancer

## Abstract

Differentiated thyroid carcinoma (DTC) is the most frequent endocrine neoplasm. Its treatment is based on surgery with consequent impact on patients’ quality of life (QoL) and aesthetic implication. The aim of the present study is to assess scar satisfaction in patients affected by DTC who underwent total or partial thyroidectomy. A comparison was also made between scar satisfaction with different subcuticular suture. Validated questionnaires have been employed during a 3-month follow-up: Patient and Observer Scar Assessment Scale (POSAS) and the Patient Scar Assessment Questionnaire (PSAQ). Eventually, the impact of thyroid cancer on QoL of patients was performed in the studied population employing the Thyroid-related patient-reported outcome questionnaire (ThyPRO) and European Organisation for Research Additionally, Treatment of Cancer—Quality of Life questionnaire-C30 (EORTC QLQ-C30). It was conducted in a single center observational study considering 74 patients respecting inclusion criteria. Overall scar satisfaction was found to improve during follow-up, reaching the best scores at 3 months from surgery. Subcuticular suture does not seem to influence the scar satisfaction. In our study male patients seem to be more satisfied, on the other hand age does not seem to influence satisfaction. Overall, the ThyPRO questionnaire and EORTC QLQ-C30 scores did not statistically differ between preoperative and postoperative suggesting a small impact of DTC in QoL.

## 1. Introduction

Thyroid carcinoma is the most frequent endocrine neoplasm (approximately 90% of cases) [1]. It is estimated that about 12,200 new cases of thyroid carcinoma were diagnosed in Italy in 2019 (about 4% of all new cases of malignant neoplasms) of which 75% were in the female sex. Its incidence has progressively increased in almost all countries during the last decades [1]. In Italy in 2019, in a population younger than 50 years old, thyroid carcinoma was the 2nd most frequent neoplasm in the female sex (5% of all neoplasms) and the 3rd most frequent in men (8% of all neoplasms) [1]. The incidence rates have also shown a marked increase, almost doubling in both genders. This rise has been attributed to an increased use of diagnostic practices leading to an earlier and more precise diagnosis [2].

More than 95% of thyroid cancer is constituted by differentiated thyroid cancer (DTC) [3]. The most common subtype of DTC is papillary (85–90%) and it is associated with the best overall survival [3].

The vast majority of thyroid cancers present as indolent thyroid nodules that sometimes could be detected incidentally by the physician during physical examination for other ENT referred symptoms, or as unexpected radiological findings. Only ∼5–10% of thyroid nodules resulted in malignancy in the general population, but males and very young or very old patients are at higher risk [4].

In order to distinguish between low- and high-risk nodules, a thorough patient history, a complete physical examination, laboratory investigations, neck ultrasonography, and, for selected patients, fine-needle aspiration (FNA), are needed [5].

A history of a rapid increase in nodule size, dyspnea, dysphagia, or hoarseness, or the development of Horner’s syndrome, albeit not specific for malignancy, are worrisome but rare findings. Concerning the neck examination, a firm, slightly mobile, and large nodule, associated with adherence to surrounding structures and the presence of lymphadenopathy, should induce the physician to consider the presence of thyroid carcinoma. Every patient suspected of thyroid malignancy should undergo fibre optic, in order to exclude the presence of vocal cord paralysis that is generally associated with advanced thyroid malignancy [6].

The treatment of thyroid carcinomas mainly consists of partial or total thyroidectomy, either associated or not with nodal dissection of the central neck compartment [7].

Thyroid surgery presents a low percentage of permanent complications. Two classical complications of thyroidectomy are due to the close anatomic proximity to the thyroid gland and other involved structures: recurrent laryngeal nerves (RLN) injuries and hypo-parathyroidism following the damage of parathyroid glands. Temporary hypoparathyroidism occurs in between 20 and 30% of cases and may be permanent in between 1 and 4% of cases. Postoperative compressive hematoma with acute dyspnea is a rare but severe complication that may result in death or severe long-term sequelae [8].

In thyroid surgery, aesthetic outcome is particularly relevant, considering that patients who undergo this kind of surgery are mostly women and young adults and that the skin incision is in a highly visible anatomic location: this aspect is frequently underestimated, considering it is less important for the patient’s global health. In order to guarantee a better cosmetic result of the scar appearance, minimally invasive approaches (MIT) for thyroid surgery have been developed over the last decade [9,10] in order to reduce tissue trauma and achieve better wound cosmetic appearance, while maintaining the same surgical outcome as traditional thyroidectomy [11,12].

Moreover, the method of skin closure contributes to the overall aesthetic outcome and patient’s satisfaction. Different methods of skin closure have been described and compared in published series with the aim of identifying the suture that can reach the best aesthetic outcome for patients [13].

Another aspect of great relevance is the evaluation of how thyroid cancer impacts the quality of life (QoL) of patients. In fact, the diagnosis of carcinomas and its perception can strongly impact patients’ everyday life, causing changes both in the physical and the psychological aspect.

The aim of this study was to compare the aesthetic appearance of cervical incision closed with three different suture techniques in patients affected by differentiated thyroid cancers (DTCs) that underwent surgery. As a secondary aim, we evaluated how the pathology and related treatment influenced QoL in these patients.

## 2. Materials and Methods

The study has been conducted as a single centre observational study in the ENT Department in Maggiore della Carità Hospital in Novara, Italy.

A series of consecutive 74 patients being affected by differentiated thyroid cancer from 1 August 2020 to 30 August 2022 who underwent cervicotomy for total or partial thyroidectomy were considered.

The inclusion criteria were:

- Age ≥ 18 years
- Diagnosis of well differentiated thyroid cancer
- Total or partial thyroidectomy
- Informed consent

The exclusion criteria were:

- Previous surgery of the neck
- Diagnosis of benign thyroid disease
- Absence of informed consent
- Lateral neck dissection

Before surgery all patients underwent ultrasound examination of the thyroid gland, fine-needle aspiration cytological examination of the thyroid nodules, thyroid specific blood tests (TSH, fT3, fT4, anti-thyroid peroxidase antibodies, antianti-thyroglobulin antibodies, calcitonin and calcium), and in some specific cases CT scan was performed.

The operation was performed through a minimal open cervical incision (length <6 cm). Meticulous haemostasis was achieved before approximation and closure of skin layers. In all patients, skin closure comes after suturing the strap muscles and subcutaneous layer with interrupted absorbable suture (Monocryl 3.0) with a single resorbable stitch. Subcuticular suturing, in the present study, varies among the populations. A single administration of prophylactic antibiotic (cefazolin 2 g) was pre-operatively administered. After the operation, patients received a standard postoperative protocol and analgesic regime.

Enrolled patients were randomly assigned through a series of random numbers generated by an electronic worksheet to have their wound closed with:

- Absorbable subcuticular suture with Monocryl 3.0
- Non-absorbable subcuticular suture with Prolene 3.0
- Steri strip^®^

The surgeon was informed of the allocation of each patient to a type of method only at the moment of closure.

The primary endpoint was the appearance of wounds in post-operative weeks assessed through two validated scar assessment scales, the Patient and Observer Scar Assessment Scale (POSAS) and the Patient Scar Assessment Questionnaire (PSAQ). Both questionnaires were submitted to all patients 10 days, 1 month and 3 months after surgery.

POSAS has been used and validated for several different types of wounds [11,14]. It is composed of two subscales, the Observer Scale (OSAS) and the Patient Scale (PSAS). OSAS is a 6-item and 10-grade Likert scale, graded from 1 (normal skin) to 10 (worst scar imaginable). PSAS is a 6-question scale exploring patients’ opinion about their scar on a 10-grade Likert scale. The higher the score; the worse the appearance of the scar, with a best possible score of 6 and a worst possible score of 60 for both subscales. Both OSAS and PSAS have a 7th item about the overall opinion graded on a 10-grade Likert scale as well.

Practitioners were trained in the use of OSAS on a series of patients before the start of this study, until they achieved a good level of agreement. Surgeons responsible for surgery could not evaluate OSAS of the patients in order to avoid possible conflicts of interest and bias in the present study. PSAS was assessed during follow-up visits with the patient in front of a mirror, while one of the observers was asking the questions [11].

The Patient Scar Assessment Questionnaire (PSAQ) with five subscales (i.e., Appearance, Symptoms, Consciousness, Satisfaction with Appearance, and Satisfaction with Symptoms) was constructed using multiple categorical response items. Each subscale consists of a set of items with 4-point categorical responses, scoring from 1 to 4 points (with 1 point assigned to the most favourable category and 4 assigned to the least favourable). Each subscale also contains a single global assessment item that is not included in the summary subscale score but is used to provide a clinically meaningful descriptor for the summary score generated and is also used for internal validation analysis [15].

Considering the second endpoint (patient’s QoL), the Thyroid-related patient-reported outcome questionnaire (ThyPRO) questionnaire is self-administered and measures QoL with 13 scales, covering physical and mental symptoms, well-being, and function as well as impact of thyroid disease on participation (i.e., social and daily life) and overall QoL. It consists of 84 items and, on average, takes 14 min to complete. Each scale ranges 0–100 with increasing scores indicating decreasing QoL (i.e., more symptoms or greater impact of disease) [16].

Moreover, the European Organisation For Research And Treatment Of Cancer—Quality of Life questionnaire-C30 (EORTC QLQ-C30) version 3.0 was used: this is a cancer-specific measure of QoL tool [17] that consists of 30 items to assess physical, role, emotional, cognitive and social functioning, global health status or QoL scales, fatigue, pain, nausea and vomiting, dyspnea, insomnia, appetite loss, constipation, diarrhoea, and financial difficulties. The scoring of the EORTC QLQ-C30 was performed according to the EORTC scoring manual [18].

Informed consent was obtained from all patients during a preoperative assessment visit. The study had ethical approval by the Ethical Committee (CE 133/22). The study was carried out in accordance with the ethical standards of the Helsinki Declaration.

The analysis was performed using SPSS for Windows (IBM Corp, Chicago, IL, USA). Normality of continuous variables was verified with the Shapiro–Wilk test (normal for *p* > 0.05). The *t*-test for paired samples and ANOVA test were used for normally distributed data. All results are reported as mean ± standard deviation (SD). Statistical significance was assumed for *p*-values < 0.05.

## 3. Results

In the examined period, 224 total or partial thyroidectomies were performed in our department. Seventy-four of them were performed in patients affected by DTC respecting inclusion criteria of the study.

Mean age at diagnosis was 57 years old [min 23–max 84 years old]. Almost 7 out of 10 patients were female (51/74).

One third of patients (23/74) who underwent surgery for DTC were considered voice professionals considering the importance of the voice in their daily work activity (teachers, singers, and so on).

The great majority of patients were affected by papillary carcinoma (83.43%); the incidence of histotype is shown in Table 1.

At the time of diagnosis, more than 90% of patients presented with a low T staging disease (T1 and T2 cancer). In this case, no one presented with T4 at diagnosis (Table 2).

In 19 patients, diagnosis of DTC was made incidentally. In these patients, thyroidectomy was performed because of the presence of hyperfunctioning thyroid goiter or deforming trachea goiter.

Sixty-three patients underwent total thyroidectomy (85.1%) and the remaining 11 lobo isthmectomy (14.9%).

**Figure 1:**
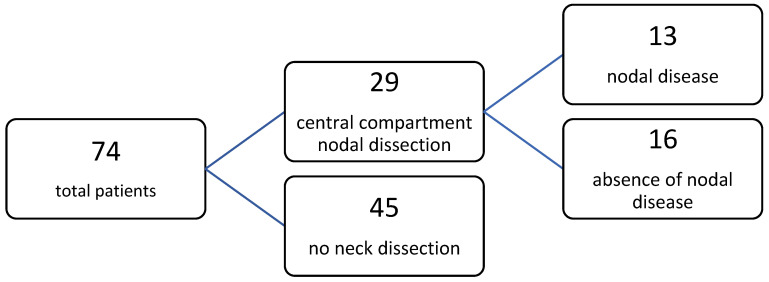
Nodal involvement of central compartment in the studied population.

In 29 cases, on the basis of the cytological result, nodule, and patients’ characteristics, a unilateral or bilateral dissection of the central compartment nodes was performed; no case of lateral neck dissection (monolateral removal of level II, III, and IV according to Robbins classification) was recommended because of nodal involvement of disease. Among 29 central neck dissections, nodal involvement was histologically documented in 13 patients (44.83%) (Figure 1).

None of the patients experienced a revision surgery because of post-surgical bleeding. No cases of scar infection were observed.

In all patients, scar assessment was performed 10 days (T1), 1 month (T2) and 3 (T3) months after surgery. Overall satisfaction of the scar was good in the entire sample since the first evaluation at 10 days. The initial scores of PSAQ were 60.8 on a maximum score of 136 points, mean values of OSAS and PSAS at first evaluation were, respectively, 11 and 14.3; it was observed that with the running time all scores employed decreased, reaching the lowest score at 3 months. These data suggest that after 3 months from the surgery there is the maximum satisfaction of the scar and consequently the lowest impact on patients’ QoL.

In Table 3 a summary of PSAQ and POSAS scores of entire population at different time of follow-up is illustrated.

**Figure 2:**
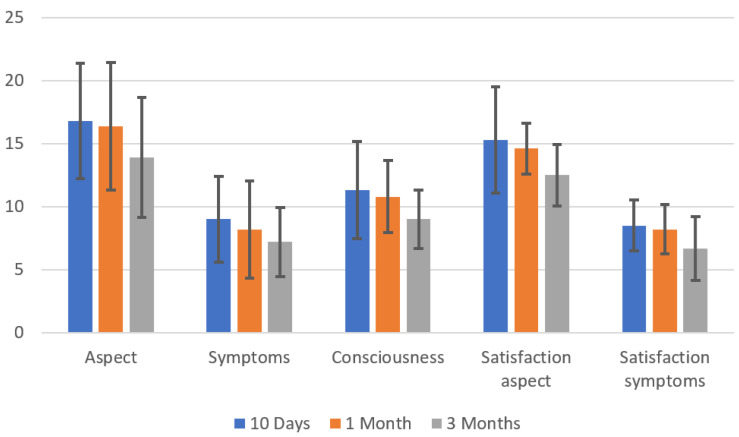
Variation, during follow-up, of five items of PSAQ.

PSAQ investigates five different items: aspect of the scar, symptoms related to scar, perception of the scar, satisfaction with the aspect of the scar, and satisfaction with symptoms of the scar. Each aspect has been analysed separately during follow up. All categories of PSAQ showed a significantly decreasing score during follow-up (Figure 2).

After the analysis of the general population, the attention was focused on the impact of scars depending on the suture techniques employed.

The three populations were compared in terms of age, gender distribution, and surgical procedure as summarized in Table 4.

**Figure 3:**
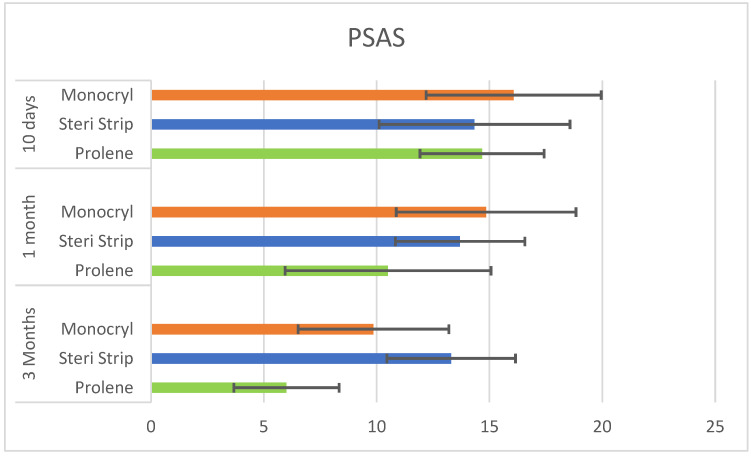
PSAS scores during follow-up in 3 different population on the basis of suture employed (*p* > 0.05).

**Figure 4:**
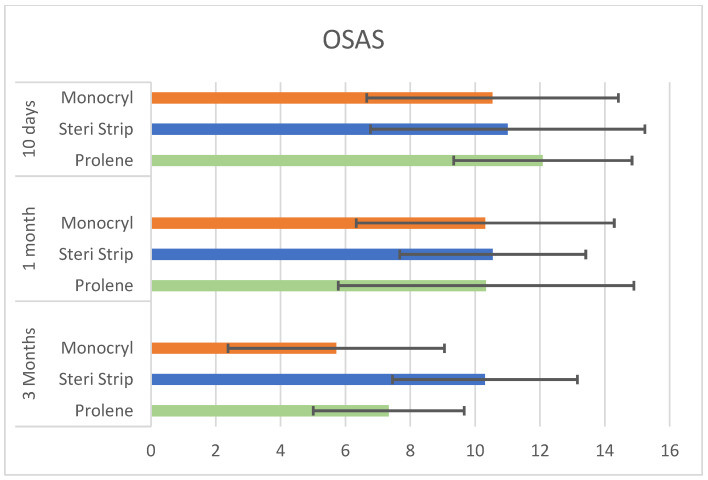
OSAS scores during follow-up in 3 different population on the basis of suture employed (*p* > 0.05).

In all three populations (identified by the subcuticular suture technique employed), a significant decrease in PSAQ, PSAS, and OSAS during follow-up was observed. On the basis of statistical analysis, the suture technique does not significantly impact on satisfaction and QoL of patients at any time of follow-up (Table 5 and Figure 3 and Figure 4).

Female gender seems to be significantly associated with a lower scar satisfaction and a heavier impact on QoL during follow-up. In fact, both OSAS and PSAS female scores were significantly higher at 1 month and at 3 months after surgery. In the PSAQ score this trend is present but does not reach statistical significance (Table 6).

As far as the influence of age on the satisfaction of scars and its impact on QoL, it was found that no statistical significance is present in PSAQ, OSAS, and PSAS among patients younger and older than 50. In both populations a significant reduction in scores during follow-up is evident (Table 7).

The impact of DTC on the QoL of patients has been investigated with the ThyPRO questionnaire and EORTC QLQ-C30 questionnaire.

ThyPRO questionnaire was administered before surgery (T0) and 1 month after surgery (T1). Thy PRO total score at T0 was 61.2 and it did not show statistical difference with T1 score of 58.9. Separately analysing each one of the 13 items, it is possible to observe that none of them presented a statistical difference between T0 score and T1 score, except for anxiety. In fact, the anxiety score decreases from a mean value of 6.37 (T0) to 3.89 (T1).

**Figure 5:**
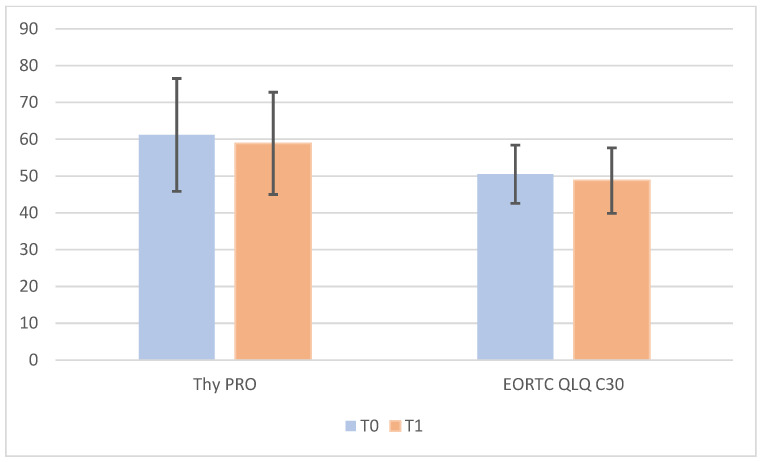
ThyPRO and EORTC QLQ-C30 variation after 1 month from surgery.

Additionally, EORTC QLQ-C30 was administered before surgery (T0) and 1 month after surgery (T1). At T0 the score was 50.5 ± 7.9. At T1 no statistical difference was recorded (48.8 ± 8.9; *p* = 0.87) (Figure 5).

## 4. Discussion

Thyroidectomy is the surgical technique of choice for many thyroid diseases, including DTC. In most cases the operation involves the complete excision of the gland for therapeutic purposes or in order to limit recurrences and simplify the follow-up [19]. The operation involves a horizontal median cervicotomy incision in an “exposed” portion of the body, which therefore may be responsible for psychological, social, and aesthetic repercussions which may, in turn, influence the general quality of life. A study conducted by Bokor et al. [20] in Germany demonstrated that scars did not affect body image in their population. On the other hand, some studies show that a significant scar at the cervical level can negatively affect the body image and therefore the quality of life [21]. All 74 patients affected by DTC operated in the Otorhinolaryngology department of the Maggiore della carità Hospital in Novara, in the observation period, were asked to fill in four different questionnaires about scar satisfaction and QoL related to Thyroid surgery/disease. All four questionnaires employed (PSAQ [22], POSAS [23], THYPRO [16], and EORTC QLQ-C30 [18]) have already been validated in the literature. They have been submitted to patients at defined follow-up intervals in order to permit a dynamic assessment of the surgical wound and of the overall quality of life. The PSAQ and POSAQ questionnaires validated for the Italian language [10] allowed us to evaluate the success and consequent satisfaction of the operated patient. The THYPRO questionnaire has been processed to investigate not only how the patient’s perception of the wound is but also the impact it can have on global quality of life (QoL). The THYPRO and EORTC QLQ-C30 evaluate various items about symptoms and QoL in patients affected by DTC. In our study conducted on 74 patients with diagnosis of DTC, the observed data demonstrated that there is a statistically significant difference (*p* < 0.05) between the PSAQ score obtained at T1 and that obtained at T3, thus confirming a better perception of the wound by the patients at the third follow up. Furthermore, analysing the scores obtained in the individual PSAQ subcategories, it can be seen that there is a statistically significant decrease in each of the five subclasses (*p* < 0.05) when comparing the score obtained at T1 with that obtained at T3.

The analysis obtained by stratifying the patients according to the type of suture has instead demonstrated how the different types of sutures (non-absorbable intradermal, absorbable sutures, and the approach of the skin flaps with Steri-strip adhesive materials) do not provide statistically significant variations (*p* > 0.05) in PSAQ and POSAQ scores, thus suggesting that patient satisfaction with the wound does not depend on the type of suture. To the best of our knowledge there are still no studies in the literature that evaluate the global PSAQ score in relation to the type of suture used; however there are studies on the evaluation of POSAQ: Teoh LY et al. reported that the use of adhesive materials versus an absorbable suture did not influence the overall satisfaction of the 96 patients included in the study [24,25]. On the contrary, some authors report how absorbable sutures provide a better aesthetic outcome and greater satisfaction compared to the flaps approaching with adhesive material [11]. Further studies, on the other hand, affirm that better outcomes are achieved if a suture with non-absorbable thread is associated with early manipulation of the wound and laser therapy [25]. It is important to remember that a well-performed suturing technique is essential to obtain a good aesthetic appearance of the scar, whatever the method chosen, and that patient satisfaction depends on many more factors beyond the appearance of the wound. Furthermore, a meticulous suture of subcutaneous layers and strap muscles is needed in order to avoid adherent scars limiting swallowing movements. Our study also compared the male and female populations: the obtained data reveal a statistically significant difference (*p* < 0.05) in POSAS scores with female gender presenting higher scores. Regarding PSAQ scores presents the same trend but, in this case, it does not reach a statistical significance.

This may depend on the fact that women pay greater attention to the healing process, and maybe have higher expectations of good wound healing. It would be interesting to understand if the higher score is only the result of greater attention to the aesthetic aspect by the female sex and the consequent fear of a possible imperfection in an area as exposed as the cervical region or if the higher score could be due to intrinsic differences in the skin in the two sexes (skin thickness, presence of lower lipid content, lower content of collagen fibres) as suggested by some authors [26].

The analysis obtained by comparing the results divided by age provided no statistically significant data (*p* > 0.05) regarding the PSAQ and POSAS scores. This data is confirmed with the study by Felix et al.: the 48 enrolled patients obtained PSAQ scores without significant differences when compared on the basis of age [27]. Despite a possible lower consideration and importance of the aesthetic factor in older individuals compared to a younger, and some physiological characteristics of the skin (greater laxity of the skin tissues, less elasticity, presence of wrinkles and skin folds which tend to mask the cervical scar), no statistically significant difference between the two populations was identified.

As far as the EORTC QLQ-C30 and ThyPRO questionnaires are concerned, no statistically significant difference was detected between pre-admission (T0) and 30 days after surgery (T1). EORTC QLQ-C30 decreased from 50.5 ± 7.9 at T1 to 48.8 ± 8.9 at T1. ThyPRO scores went from 61.2 ± 6.9. (T0) to 58.9 ±7.1 (T2). The fact that patients’ QoL does not vary before and after surgery could be explained by the fact that the majority of DTC are diagnosed in early stage, sometimes incidentally and in consequence no or light symptoms influence life of patients. In fact, as shown in our analysis the only item that significantly decreases after surgery is anxiety. This is justified by the malignant nature of the pathology and the consequent relief after surgery with the perception of recovery from disease.

Our study has allowed us to evaluate how in the analysed populations the general perception of injury is good and it improves with the running time, reaching the best scores after 3 months. Scar perception can change in relation to gender, but age and the type of suture performed seem to be irrelevant.

Our research presents some limitations: first of all, patients involved in the study are Caucasian and in consequence our data do not consider the possible influence of other cultural factors regarding the scar perception in an exposed anatomical site. Moreover, in our population it was not considered a psychological evaluation which could evidence depressive disorder or dysmorphophobic disorder potentially influencing scar perception.

It would be interesting to stratify the population, also on the basis of the profession, dividing for example the patients into those who perform a “public” job in comparison with those who have more limited social relations.

Another further starting point would be to compare patients with different cultural levels, thus trying to understand if the level of education can modify the evaluation in any way. Finally, with a larger sample, the population could be divided according to ethnicity or phenotypic characteristics, and evaluation of how this correlates with the quality of healing process and the perception of the wound could be explored.

Eventually, scar cosmesis perception after transcervical is also a relevant issue to be considered, even more so in the era of remote access robotic or trans-oral approaches.

## 5. Conclusions

In conclusion, scars associated with thyroid surgery are well accepted by the patients. Patients seem to be satisfied with surgical scars even at the first postoperative follow up and their satisfaction seems to improve over time. Subcuticular suture technique does not seem to influence scar satisfaction. Female gender seems to be significantly associated with a lower scar satisfaction and a heavier impact on QoL during follow-up; however, age does not seem to influence scar satisfaction. In our study the diagnosis of thyroid carcinoma does not seem to influence the patient’s QoL. All these data could suggest that even if thyroid surgery-related scars are clearly visible, the patient’s satisfaction and the low impact on QoL permit the surgeon to safely employ the conventional neck approach rather than mini-invasive or robotic assisted surgeries that are reported to be more aesthetic but technically complex, expensive, and time consuming.

## Figures and Tables

**Table 1:** Incidence of histotype in studied population (with the term “others” we indicate patients with both papillary and follicular foci).

Histology	N° (%)
Papillary carcinoma	61 (82.43%)
Follicular carcinoma	5 (6.67%)
Others	8 (10.81%)

**Table 2:** T staging at diagnosis.

T Staging		N° (%)
T1	a	43 (58.11%)
	b	13 (17.57%)
T2		12 (16.21%)
T3	a	6 (8.11%)
	b	0
T4		0

**Table 3:** PSAQ and POSAS scores of entire population at different time of follow-up.

	10 Days (T1)	1 Month (T2)	3 Months (T3)	*p* Value
PSAQ [34–136]	60.8 ± 8.3	59.5 ± 6.8	49.3 ± 6.1	<0.001
OSAS [5–50]	11 ± 2.3	11.2 ± 2.4	11.2 ± 1.8	0.004
PSAS [6–60]	14.8 ± 3.3	14.1 ± 2.9	10.9 ± 2.8	0.01

**Table 4:** Mean age, gender distribution, and surgical procedure in the three populations.

Variable		Prolene	Steri Strip^TM^	Monocryl	*p* Value
Gendre	F	8 (32%)	7 (24.14%)	8 (40%)	0.4953
	M	17 (68%)	22 (75.86%)	12 (60%)	
Age	Mean value	57	57.45	52.1	0.4073
	SD	12.23	16.54	14.35	
Surgery	Toal thyroidectomy	22 (88%)	24 (82.76%)	17 (85%)	0.8642
	Partial thyroidectomy	3 (12%)	5 (17.24%)	3 (15%)	

**Table 5:** PSAQ scores during follow-up in 3 different population on the basis of suture employed.

Time from Surgery	Suture	PSAQ	SD	*p* Value
10 days	Prolene	58.75	10.72	0.71
	Steri Strip	61.72	10.25	
	Monocryl	61.47	9.6	
1 Month	Prolene	55.17	11.99	0.65
	Steri Strip	60.38	12.62	
	Monocryl	56.54	13.87	
3 Months	Prolene	43.67	11.93	0.71
	Steri Strip	51.50	13.6	
	Monocryl	50.14	15.87	

**Table 6:** PSAQ, PSAS, OSAS scores variations during follow-up in male and female populations.

	Time from Surgery	M	F	*p* Value
PSAQ	10 Days	56.94 ± 10.83	63.00 ± 11.3	0.88
	1 Month	50.20 ± 11.31	61.32 ± 10.92	0.23
	3 Months	39.00 ± 10.42	53.47 ± 11.1	0.35
OSAS	10 Days	11.19 ± 2.16	10.90 ± 1.82	0.67
	1 Month	7.60 ± 1.85	11.68 ± 1.94	0.03
	3 Months	5.40 ± 1.62	9.20 ± 2.19	0.002
PSAS	10 Days	12.50 ± 2.54	16.07 ± 2.16	0.71
	1 Month	10.40 ± 2.14	15.00 ± 1.75	0.031
	3 Months	9.00 ± 1.94	12.13 ± 1.62	0.011

**Table 7:** PSAQ, PSAS, OSAS scores variations during follow-up in populations younger and older than 50.

	Time from Surgery	≤50	>50	*p* Value
PSAQ	10 Days	65.33 ± 10.88	58.60 ± 11.03	0.03
	1 Month	60.92 ± 11.34	56 ± 11.26	0.36
	3 Months	59.6 ± 10.9	46.6 ± 10.82	0.49
OSAS	10 Days	10.33 ± 1.87	11.53 ± 2.12	0.3
	1 Month	10.33 ± 2.33	10.45 ± 2.38	0.95
	3 Months	6.6 ± 1.75	8.8 ± 2.21	0.16
PSAS	10 Days	16.4 ± 2.84	14.3 ± 2.63	0.28
	1 Month	16.08 ± 2.57	12.05 ± 2.83	0.01
	3 Months	11 ± 1.98	11 ± 1.47	0.36

## Data Availability

All data are available on request.
